# An Assay to Monitor HIV-1 Protease Activity for the Identification of Novel Inhibitors in T-Cells

**DOI:** 10.1371/journal.pone.0010940

**Published:** 2010-06-03

**Authors:** Brett J. Hilton, Roland Wolkowicz

**Affiliations:** Department of Biology, San Diego State University, San Diego, California, United States of America; University of Pittsburgh, United States of America

## Abstract

The emergence of resistant HIV strains, together with the severe side-effects of existing drugs and lack of development of effective anti-HIV vaccines highlight the need for novel antivirals, as well as innovative methods to facilitate their discovery. Here, we have developed an assay in T-cells to monitor the proteolytic activity of the HIV-1 protease (PR). The assay is based on the inducible expression of HIV-1 PR fused within the Gal4 DNA-binding and transactivation domains. The fusion protein binds to the Gal4 responsive element and activates the downstream reporter, enhanced green fluorescent protein (eGFP) gene only in the presence of an effective PR Inhibitor (PI). Thus, in this assay, eGFP acts as a biosensor of PR activity, making it ideal for flow cytometry based screening. Furthermore, the assay was developed using retroviral technology in T-cells, thus providing an ideal environment for the screening of potential novel PIs in a cell-type that represents the natural *milieu* of HIV infection. Clones with the highest sensitivity, and robust, reliable and reproducible reporter activity, were selected. The assay is easily adaptable to other PR variants, a multiplex platform, as well as to high-throughput plate reader based assays and will greatly facilitate the search for novel peptide and chemical compound based PIs in T-cells.

## Introduction

The Human Immunodeficiency Virus (HIV) was identified as the causative agent of Acquired Immuno Deficiency Syndrome (AIDS) in 1983 [1] and has since resulted in over 33 million deaths (UNAIDS/WHO Report 2009). HIV, a lentivirus belonging to the *Retroviridae* family of viruses, has a ∼9.6kb diploid RNA genome, encoding nine genes and at least 15 protein products, resulting from various mechanisms including alternative splicing, ribosomal frame shifting and proteolytic cleavage of the proteome [2], [3].

In spite of the lack of progress in the field of vaccine development against HIV, three decades of research has resulted in various antivirals. The antiviral drugs developed so far target the viral proteins, including Protease (PR), Reverse Transcriptase, Integrase and Envelope (fusion process), or the cellular receptors involved in viral entry. In total, about 32 inhibitors (11 targeting PR) have been approved by the FDA, since the first Protease Inhibitor (PI), Saquinavir was launched in 1995 [4]. Inhibitors, supplied as a cocktail of drugs of three or more inhibitors through Highly Active Anti-Retroviral Therapy (HAART) have resulted in a drastic reduction in the number of AIDS-related deaths [5].

HIV PR, a 99 amino acid aspartyl protease, is responsible for the post-translational cleavage of the HIV Gag and Gag-Pol poly-protein precursor proteins in the host [6], [7]. PR targets all the proteolytic cleavage sites within the viral proteome, except the envelope gp120/gp41 boundary [8], [9], which is cleaved by the host pro-protein convertase furin or furin like-proteases [10], [11]. The catalytic core of PR consists of the Asp, Thr, Gly triad, and mutations at Asp25 have been demonstrated to result in the loss of catalytic activity [12]. PR also has autocatalytic properties, enabling its own removal from the precursor poly-protein, following dimerization of the Gag-Pol precursor protein [13]. PR activity is absolutely necessary for the production of replication-competent virus, thus making processing by PR an obvious target for anti-retroviral drug development [14]. One of the earliest identified inhibitors of PR activity was Pepstatin A, an aspartyl protease inhibitor, which results in the impaired processing of HIV proteins, leading to the production of aberrant virions [15], [16].

The majority of assays developed so far, in order to screen for PIs, have been designed *in vitro* [17]–[19] in bacterial [20], [21], or yeast cells [22]. The lack of assays for PR activity in mammalian cells is, in general, due to the cytotoxic effects of PR [23]. The recently developed mammalian cell-based assays [24], [25], although invaluable, may not provide accurate evaluation of the inhibitory effects of putative novel drugs on PR as they are not performed in the natural environment of HIV infection, T-cells. Moreover, most of the assays are not adaptable for efficient and reliable high-throughput screening. The growing need for effective assays, which can aid in drug discovery, prompted us to develop a simple yet rapid and straightforward method for monitoring PR activity. The assay, developed in T-cells, will thus facilitate the search for novel PIs/competitors in a cell type naturally infected by HIV.

The assay described here is based on the classical Gal4-UAS system, a system broadly utilized for gene expression studies [26]. The yeast Gal4 protein represents a prototypic transcription factor consisting of two separate domains: an N-terminal DNA-binding domain (DBD: amino-acids 1–147) and a C-terminal trans-activation domain (TAD: amino-acids 768–881). The Gal4 protein binds to the consensus Upstream Activation Sequences (UAS) *via* its DBD and activates transcription of downstream genes through its TAD. However, when the two Gal4 domains are separated, neither of them can function as a transcription factor by itself [27].

Murray *et al.*, in 1993 first demonstrated the ability of HIV-1 PR fused within Gal4 domains to autocatalytically remove itself, leaving behind the two non-functional domains of Gal4 [22]. However, in the presence of PI, or when the PR/Gal4 fusion protein is mutated at the catalytic site, the fusion protein remains intact, retaining its ability to bind to UAS and subsequently activate transcription. This concept has also been applied for the characterization of the autocatalytic activity, of other proteases like the 3C protease of coxsackievirus B3 [28]. We exploited this property of the Gal4 system to express a reporter gene dependent on PR activity, the expression of the reporter gene being inversely proportional to the autocatalytic activity of PR, thus serving as the basis for our assay.

The assay described in this paper is based on the expression of the PR/Gal4 fusion in an inducible manner via a Tet-On system (adapted from Clontech), thus drastically reducing the possible toxic side-effects of PR. The reverse tetracycline transactivator (rtTA) used in this system allows for the induction of PR/Gal4 expression by the addition of tetracycline (Tet) or doxycycline (Dox), only when needed. eGFP, the sensor for PR activity, will be expressed only when PR/Gal4 expression is induced in the presence of a PI. Most importantly, all the assay elements have been stably expressed in mammalian cells using retroviral technology. Finally, we established T-cell lines from clones with the highest sensitivity showing robust, reliable and reproducible behavior. This is the first time a cell-based assay for monitoring PR activity has been developed in T-cells, the natural *milieu* of HIV infection, thus providing an efficient way to search for novel inhibitors/competitors of PR, which could lead to the development of new therapeutics against HIV.

## Results

### A Gal4/PR fusion protein that activates reporter gene expression only when inhibited

**Figure 1:**
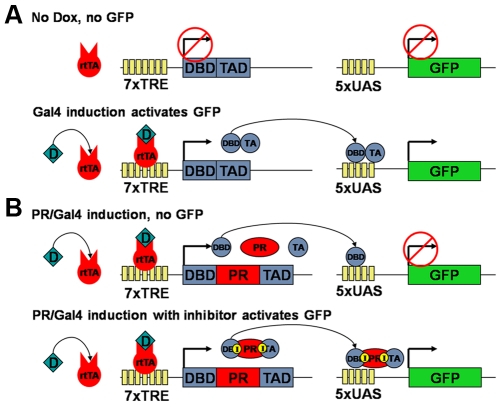
Assay overview. (**A**) Wild type Gal4 as control, without Dox. In the absence of Dox, rtTA can not bind to the Tet-Responsive Element (TRE) and consequently, there is no eGFP expression from the reporter construct. In the presence of Dox, rtTA binds to TRE and induces eGFP expression. (**B**) The PR/Gal4 fusion-based system. In the presence of Dox, PR/Gal4 is expressed; however, its autocatalytic activity results in the separation of the Gal4 domains, resulting in lack of eGFP expression. However, in the presence of PI, the PR/Gal4 fusion remains intact, resulting in the induction of eGFP expression. The same result is expected with an inactive mutant PR, but without the need of inhibitor.

**Figure 2:**
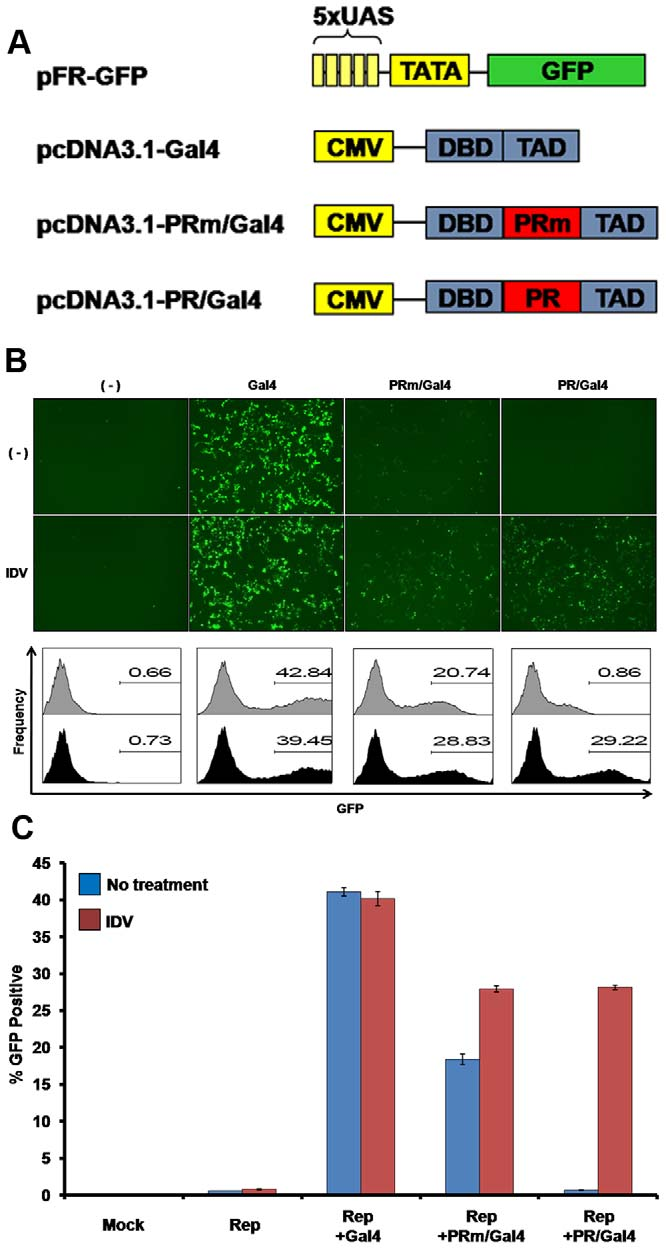
Transient expression of the assay components in HEK293T cells. (**A**) Vectors used for the transient expression of the assay. The constructs include the eGFP reporter vector and the mammalian expression vectors for Gal4, PRm/Gal4 and PR/Gal4. (**B**) Top Panel: Fluorescence microscopy of cells 24 hours post transfection with reporter alone (-), or together with either the Gal4, PRm/Gal4, or PR/Gal4 vectors with or without PI. Bottom panel: Cells were analyzed by flow cytometry at 24 hours post transfection. Percentage of eGFP expressing cells is indicated for each treatment. Black and dark grey histograms are with or without 10µM IDV. (**C**) Quantification of eGFP expression, indicative of PR inhibition, based on flow cytometry data. Results shown are average of three independent experiments.

We established a stable T-cell line, which will provide the basis for an assay that facilitates monitoring of HIV-1 PR activity, as outlined in **Fig. 1**. For this purpose, we first verified that each of the elements of the assay responded as expected in a transient manner, prior to proceeding with the establishment of stable cell lines. A reporter vector, referred to as pFR-eGFP, containing a 5xUAS Gal4 responsive element upstream of a minimal CMV (mCMV) promoter followed by the eGFP gene (**Fig. 2A**) was transiently expressed in adherent HEK293T cells and these cells showed little to no background (**Figs. 2B and 2C**). Next, we co-transfected the reporter vector with pcDNA3.1-Gal4 (**Fig. 2A**), a construct based on the mammalian expression vector, pcDNA3.1. The Gal4 gene utilized here, minimal Gal4, encodes only the DBD and TAD segments of Gal4. Even though other variants of Gal4, such as Gal4VP16, are capable of higher levels of induction under the UAS control, minimal Gal4 allows for insertion of a proteolytic enzyme within its well-characterized domains, while still retaining high level of transcriptional activity. As expected, co-transfection of the reporter vector with pcDNA-Gal4 led to a dramatic induction of eGFP expression in 293T cells (**Figs 2B and 2C**).

Next, we corroborated that the insertion of the PR sequence within Gal4, between DBD and TAD, does not disrupt the transcriptional activity of Gal4. To test this, we first introduced PR D25A, an inactive version of PR (PRm), between the Gal4 domains. In addition to the PR sequence, based on the HXB2 HIV-1 genome, 22 upstream and 32 downstream amino acids (to include the PR cleavage sites) was introduced in between the Gal4 DBD and TAD (**Fig. 2A**). As PR D25A has been shown to lack catalytic activity, it should not be able to separate the domains, and thus disrupt the ability of the DBD and TAD to work in conjunction to activate eGFP expression. As expected, reporter and pcDNA-PRm/Gal4 co-transfection in 293T cells resulted in significant eGFP expression (**Figs. 2B and 2C**). Although the induction of eGFP expression by the PRm/Gal4 fusion was lower than that of Gal4 alone, the level of activation was clear, corroborating that the insertion of the specific PR sequence ‘*per se*’ does not have any major impact on Gal4 function.

The PRm sequence was then substituted with the wild-type, active PR sequence. This sequence contained the exact same additional 22-upstream and 32-downstream amino acids of PR, but retained the wild-type aspartic acid residue at position 25. Importantly, co-transfection of reporter and wild-type PR/Gal4 fusion vectors led to a significant reduction in eGFP expression as compared to PRm/Gal4 (**Figs. 2B and 2C**). Finally, it was crucial to verify that addition of PI would reconstitute the ability of PR/Gal4 to activate the reporter and thus result in eGFP expression. For this purpose, HEK293T cells were pre-treated with 10µM Indinavir (IDV, chosen as an example of FDA-approved PI) prior to transfection of reporter and PR/Gal4 vectors. While control cells or cells incubated with DMSO alone lacked eGFP expression, cells incubated with 10µM IDV showed a drastic induction of eGFP (**Figs. 2B and 2C**).

### Design of lentiviral constructs for the inducible expression of PR in T-cells

**Figure 3:**
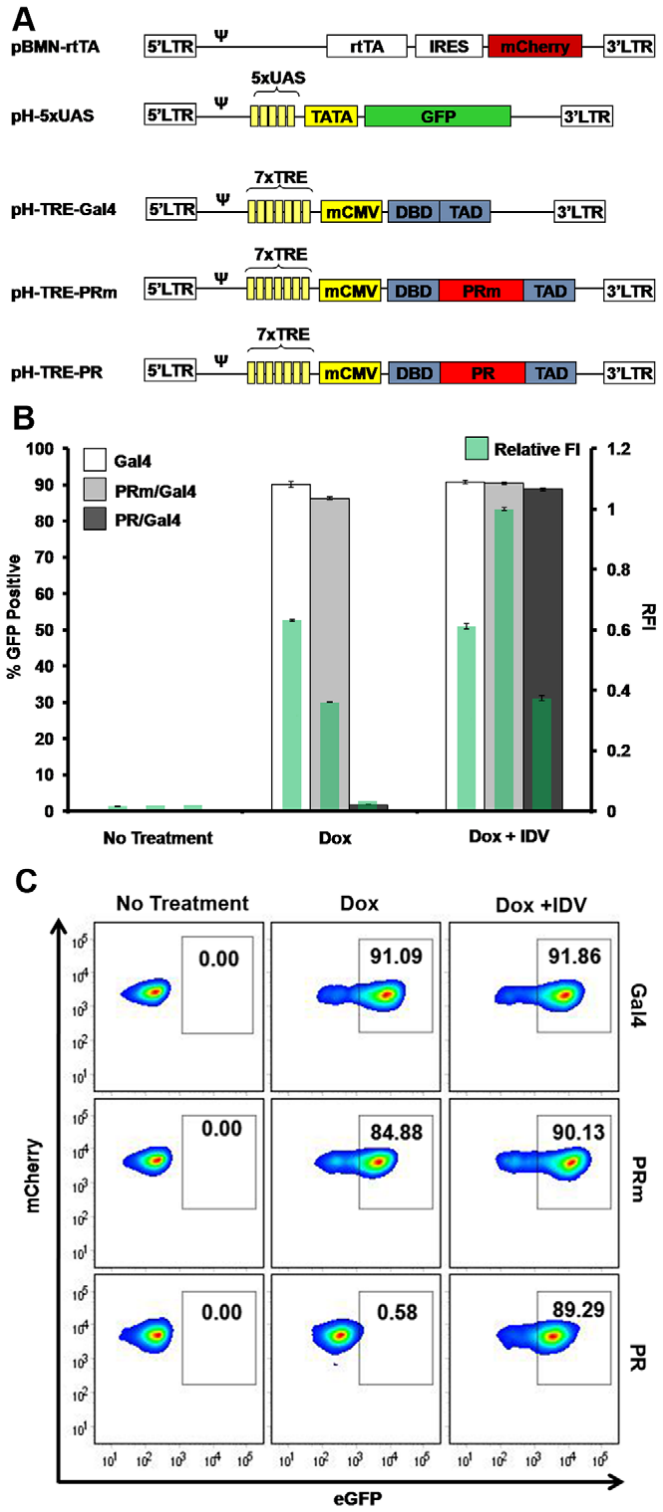
Establishment of a clonal T-cell line stably expressing inducible assay elements. (**A**) Constructs utilized to generate infectious particles for the transduction of SupT1 cells with the various assay elements (the rtTA, eGFP reporter, and Gal4-based vectors: Gal4, PR/Gal and PRm/Gal4). (**B**) Flow cytometry analysis of selected clones. Clones expressing the elements of the assay were analyzed with no treatment, with 1µg/mL Dox, or with 1µg/mL Dox and 10µM IDV. (**C**) Quantification of eGFP expression (left axis and larger bars) and the relative fluorescence intensity (RFI) of each sample (right axis and green bars within larger bars) of clones treated as indicated. The RFI was calculated by normalizing green mean fluorescence intensity (MFI) to the brightest MFI observed (PRm with Dox + IDV).

Next, we wanted to test if these results could be reproduced in T-cells, a cell-type that represents a more natural *milieu* for HIV-1 infection. For this purpose, we utilized retroviral technology [29] to stably express the elements of the assay in SupT1 cells, a T-cell line easily infected by HIV-1 and broadly utilized in HIV-1 studies. The UAS reporter element and Gal4 or PR/Gal4 fusions were transferred into lentiviral vectors. First, the reporter sequence was inserted into an HIV-based self-inactivating vector, with most of the 3′ LTR U3 sequence deleted for safety reasons as well as to ensure that the reporter activity is based only on the 5xUAS element of the reporter and not the viral promoter. In addition, this ensures that there will be no background reporter activity in the absence of inhibitor. The reporter lentiviral vector is referred to as pH-5xUAS-eGFP (**Fig. 3A**). As mentioned before, we wanted to create a system that enabled inducible expression of the PR/Gal4 fusions in order to circumvent the possible cytotoxicity of PR when expressed in mammalian cells.

In order to obtain an inducible cell line, we utilized the tetracycline inducible (Tet-On) system (adapted from Clontech). In this system, rtTA binds to and activates expression from the Tetracycline Response Element (TRE) only in the presence of Tet or Dox. For this purpose, two retroviral/lentiviral vectors were constructed. One of the vectors carries the rtTA element coupled to mCherry through an Internal Ribosome Entry Site (IRES), allowing corroboration of rtTA expression based on red fluorescence (pBMN-i-rtTA plasmid in **Figure 3A**). The second vector harbors a 7x TRE element upstream Gal4, PRm/Gal4, or PR/Gal4: pH-TRE vectors in **Figure 3A**).Viral particles were produced as described in the ‘Methods’ section using the reporter, and rtTA plasmids, and the viral supernatant was collected and used to transduce SupT1 cells. A stable cell line expressing these elements was then transduced with viral particles carrying Gal4 (pH-TRE-Gal4), PRm/Gal4 (pH-TRE-PRm/Gal4) or PR/Gal4 (pH-TRE-PR/Gal4). When SupT1 cells were transduced with lentiviral particles containing the reporter vector alone, no detectable eGFP expression was observed and similar results were obtained with virus encoding inducible Gal4 alone (**Fig. 3B**). Importantly, when cells were co-infected with virus containing reporter, rtTA and inducible Gal4, or inducible PRm/Gal4, eGFP expression was undetectable. However, when these cells were treated with 1µg/mL of Dox, eGFP expression was clearly induced (**Fig. 3B**), confirming the inducibility of the system and hence PR expression in T-cells.

Importantly, the TRE-PR/Gal4 population showed very low eGFP expression, even after the addition of Dox. However, when these cells were treated with 10µM IDV, a large induction of eGFP expression was observed (**Fig. 3B**), thus validating the assay and the utility of eGFP as a biosensor for the inhibitory activity of antivirals, at least IDV, on PR in SupT1 cells.

### Generation and selection of clonal stable cell lines with the best sensitivity

Previous experiments were performed with non-clonal cell populations. The goal of the assay, however, was to design an assay in T-cells with a definitive and robust readout. Therefore, we aimed to purify and amplify specific clones from the population that displayed the lowest degree of background and the highest level of eGFP expression in response to the appropriate treatment.

For this purpose, the initially transduced cell populations harboring rtTA, eGFP reporter construct and either Gal4, PR/Gal4 or PRm/Gal4 were pre-incubated with 10µM IDV. Cells were then sorted 24 hours later based on eGFP expression in order to enrich for those cells with an activatable reporter and an inducible transcription factor. Seven days later, after the eGFP from the previous activation disappeared, one more round of sorting was performed under identical conditions, followed by a final round of sorting at day 14. However, in this final round, sorting was performed to isolate cells with no eGFP expression (i.e. cells with little to no background), which resulted in a cell population that was up to 80% positive for eGFP after activation, with nearly zero background (**Fig 3C**).

Lastly, individual cells from the sorted populations (negative for eGFP expression when untreated), were plated in a 96 well-plate format. Single clones were expanded to obtain clonal cell lines, which were subsequently activated using the previously described conditions (1µg/mL of Dox and 10µM IDV) and screened for the highest response to appropriate conditions (data not shown). Individual clones with maximal eGFP activation were selected for each of the inducible elements; Gal4, PRm/Gal4 and PR/Gal4. The selected clones exhibited almost 100% activation with nearly undetectable background (**Fig 3C**). These clones were then expanded and further analyzed in the following experiments.

### Optimization and kinetics of the assay

**Figure 4:**
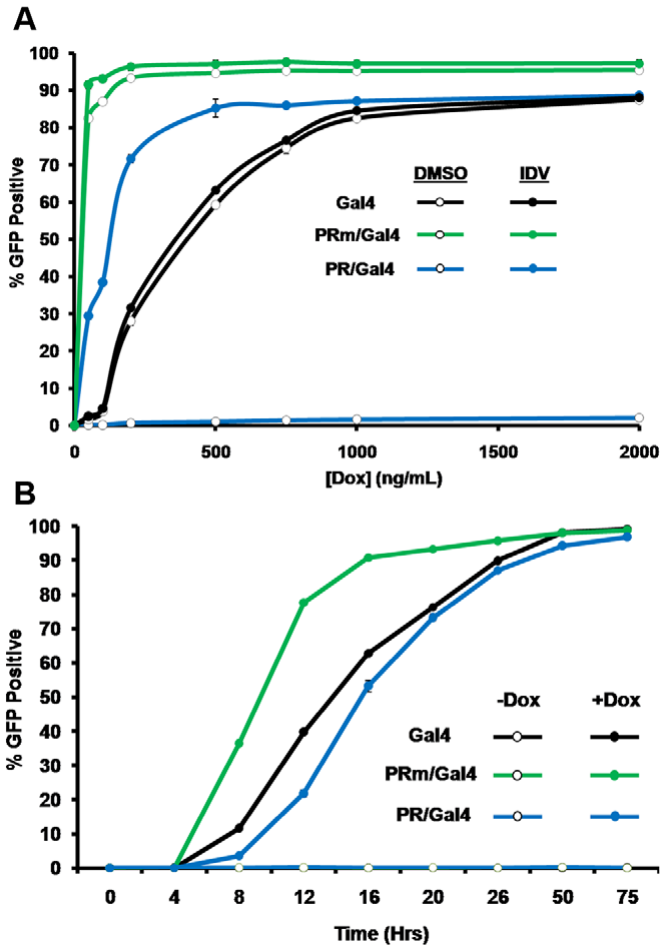
Determination of the optimal conditions for the screening of active PIs. Clonal SupT1 cells harboring inducible Gal4, PRm/Gal4, or PR/Gal4 were activated in a 96 well-plate format under various conditions. (**A**) Doxycycline titration. Cells were pre-incubated with either DMSO or 10µM IDV and then either left untreated, or activated with 50, 100, 200, 500, 1,000, or 2,000ng/mL of Dox. Cells were analyzed by flow cytometry to determine the number of eGFP positive cells. (**B**) Time course analysis of eGFP induction in response to Indinavir with or without Dox. Cells were pre-incubated with 10µM IDV and then either left untreated, or activated with 1µg/mL Dox. Cells were analyzed 4, 8, 12, 16, 20, 25, 50 or 75 hours after activation by flow cytometry.

In order to optimize the assay for maximal induction of Gal4, PRm/Gal4 or PR/Gal4, we analyzed the effect of increasing levels of Dox in the assay. Cells treated with 0, 50, 100, 250, 500, 750, 1,000, and 2,000 ng/mL Dox, were either treated with DMSO or 10µM IDV and analyzed 32 hours later (**Fig. 4A**). TRE-Gal4 cells reached saturation with Dox at about 1,000ng/mL irrespective of the presence of IDV. Surprisingly, TRE-PRm/Gal4 was induced at lower levels of Dox, and reached saturation at around 250ng/mL in both IDV-treated and untreated cells. Again, as with transiently transfected HEK293T cells, TRE-PR/Gal4 had little to no detectable eGFP expression at any given Dox concentration in the absence of 10µM IDV. However, pre-incubation of TRE-PR/Gal4 cells with 10µM IDV showed maximal eGFP induction at around 500ng/mL Dox. This trend was similar to that observed in the transient expression experiment.

Next, in order to determine the optimal time point for the analysis of eGFP expression in the assay, cells were activated with Dox and analyzed by flow cytometry at various time points. Cells were treated either with DMSO alone as vehicle control, or 1µg/mL Dox and 10µM IDV (**Fig. 4B**). DMSO-treated cells showed no fluorescence throughout the experiment. However, Dox-activated cells incubated with IDV showed initial induction of eGFP expression at about 8 hours and reached nearly 100% at about 50 hours post-induction.

### Assay response to other PIs

**Figure 5:**
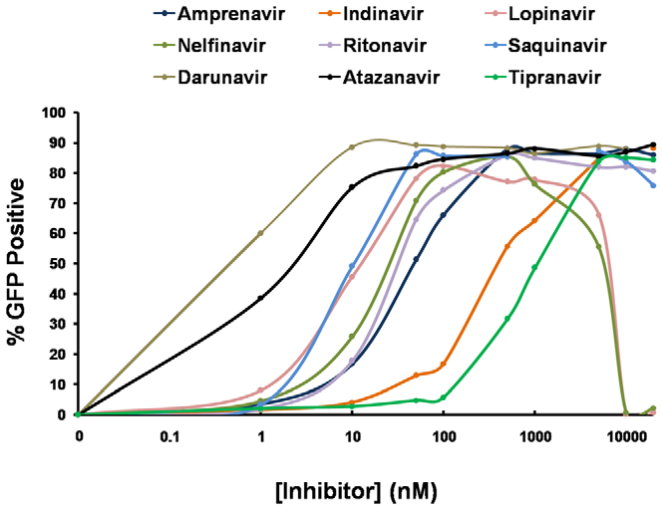
Assay response to FDA-approved PIs. The selected SupT1 clone expressing rtTA, the 5xUAS-eGFP reporter and an inducible PR/Gal4 were incubated with various concentrations of Amprenavir, Atazanavir, Darunavir, Lopinavir, Nelfinavir, Ritonavir, Saquinavir, or Tipranavir, as well as Indinavir, and then activated with 1µg/mL Dox and analyzed 50 hours later by flow cytometry.

Finally, a preliminary screening was performed with eight other FDA-approved PIs to ensure that the assay is not specific for IDV and is sensitive to other inhibitors as well. For this purpose, cells were incubated with DMSO alone, as described above (data not shown), or with increasing concentrations of Atazanavir, Amprenavir, Darunavir, Nelfinavir, Lopinavir, Ritonavir, Saquinavir or Tipranavir, as well as IDV (**Fig. 5**). We have chosen a broad concentration range (from 1nM to 20µM) including the ones most commonly used in cell culture as well as the low ranges that are typically not active in less sensitive assays.

From our data, it is clear that Darunavir and Atazanavir had the strongest inhibitory effects on PR activity, resulting in eGFP expression at just 1nM, while IDV and Tipranavir, showed the weakest inhibitory effects, nevertheless achieving full induction of eGFP expression at about 5µM. Interestingly, all the tested PIs resulted in increasing eGFP expression levels with increasing inhibitor concentrations, up to 20µM, the maximal level tested. However, Lopinavir and Nelfinavir resulted in cell death at a concentration of about 1µM, with significant decline in the number of eGFP positive cells, as was the case with Saquinavir at 20µM. Overall, all the inhibitors tested showed significant inhibitory effect on PR activity and resulted in the activation of eGFP expression in our SupT1 clone.

## Discussion

In spite of the existence of various bacterial or yeast cell based HIV PR assays, only few are mammalian cell-based; of these, none are in a cell type actually infected by HIV-1. In order to achieve high-throughput screening of HIV PIs in a relevant setting with a high readout, we have produced a stable T-cell line that can be used as a sensitive indicator of HIV-1 PR activity. We expect this assay to greatly facilitate biological screenings, using peptide or chemical compound based libraries, and thus result in the discovery of novel PIs in T-cells in a straightforward and prompt manner.

In the assay, the PR/Gal4 fusion was able to activate the reporter vector in HEK293T cells in transient experiments only in the presence of the inhibitors, as expected. This proved that the assay functions as anticipated, and was thus transferred into retroviral vectors for stable expression in T-cells. Stable cell lines exhibited little to no detectable levels of background eGFP expression, a critical factor for the efficient and reliable utilization of the assay in high-throughput screenings. The Off/On inducible expression of the PR fusion protein proved to be effective and highly Dox dependent. Importantly, the inducible system results in the expression of PR only at the time of the screening process, thus preventing their possible cytotoxic effects prior to that, as demonstrated by the viability of the clonal cell line at least for the time period analyzed.

Additionally, the mean fluorescence intensity of eGFP in the activated clones steadily increased, as inhibited cells continued to accumulate higher levels of eGFP, making the cells capable of detecting extremely low levels of PR inhibition over time, as seen in **Fig 5**.

An unexpected observation was the transactivational activity of the PR D25A mutant variant described as catalytically inactive (PRm). Based on previous reports, the insertion of the PRm sequence was expected to have little effect on the ability of Gal4 to transactivate the reporter plasmid. While the percentage of positive cells with or without IDV remained the same, surprisingly, the relative fluorescence intensity (RFI) increased significantly with the addition of inhibitor. Thus, the further increase in fluorescence intensity was unexpected, as full activation was anticipated to be independent of PI addition. This was consistent with the results obtained in the transient experiments as well. These findings hint at the possibility that PR D25A is not a completely inactive form of PR or possibly may be targeted by an unknown cellular protease. In either case, this observation corroborates the sensitivity and usefulness of this assay, not only for the analysis of HIV PR activity, but also for the study and characterization of other PR variants, wild-type or mutant, and of other proteases in general, viral as well as cellular. The potential partial activity of PR D25A in T-cells as observed in our assay has not been previously described and certainly warrants future research. This observation also re-enforces the fact that the assay, developed in T-cells, may provide a more accurate environment for antiviral drug discovery against HIV.

Importantly, the elements of the assay, expressed in the human T-cell lymphoblastic lymphoma cell line, SupT1, can easily be transferred into other relevant cell types known to be vital for the establishment of HIV infection, such as macrophages and dendritic cells. Furthermore, the assay can be used to study the molecular evolution of PR, by replacing the wild-type PR sequence with PR mutants arising in patients, untreated and HAART-treated alike. Moreover, the assay can be used to determine the appearance of drug resistance.

The assay is designed to facilitate the efficient screening for novel inhibitory chemical compounds or peptides in a simple flow cytometry-based platform. The lack of background noise in the assay and clear distinction between negative and positive hits will allow screening to be conducted with high efficiency and minimal false positives. Additionally, the eGFP-based readout will allow screening of millions of candidates (through rational or non-rational based approaches) in a single experiment, with extreme sensitivity. The inhibition by other FDA-approved PR inhibitors proved that the applicability and sensitivity of the assay is not limited to IDV, validating once again the ability to utilize eGFP expression as a biosensor for PR activity in SupT1 cells. In this respect, it is worth mentioning that Tipranavir, although considered one of the most potent inhibitors, demonstrated lower potency in our assay. While this fact may be of biological significance, Tipranavir still fully activated the reporter gene at 5µM. Therefore, all FDA-approved PIs had inhibitory effects in our assay, at concentrations well within the range of testable concentrations for screening. Moreover, while we had chosen 10µM IDV for our proof-of-concept experiments, the assay was clearly shown to be sensitive at lower concentrations as well.

While genotypic and phenotypic testing for HIV resistance to antiretroviral drugs has been proven useful for individual patient treatment management, the primary goal of the assay described here is not to calculate IC50s of the FDA-approved drugs, but to find new inhibitors. However, due to its high sensitivity, the assay could be used to analyze the potency range of a specific drug, at least as defined by the relative fluorescence intensity and percentage of fluorescent cells. While IC50 values described in literature vary greatly depending on the method in which they were tested, our results show a range within those observed in literature [30]–[34]. Figure 5 shows that the approximate concentration to reach a 50% relative fluorescent intensity for drugs such as Atazanavir, Lopinavir, Indinavir or Tipranavir is 1-to-10nM, 10-to-50nM, 100-to-500nM and 500-to-1000nM, respectively. Based on the results, a screen utilizing the assay developed here would be optimally conducted at drug concentrations up to 1µM.

Overall, we have developed an assay that can be easily adapted to further enhance its high-throughput capacity in a flow cytometry as well as luminescent-plate reader based format. The assay, developed in T-cells for the first time, will greatly promote the screening efforts for novel HIV PIs.

## Materials and Methods

### Cloning and Vector Construction

Gal4, PR/Gal4 and PRm/Gal4 sequences were amplified by PCR for the production of the transient expression vectors pcDNA-Gal4, pcDNA-PR/Gal4 and pcDNA-PRm/Gal4, respectively. For this purpose, the constructs pMA236, pHP236 and pHP236m [22] were used as templates together with the Gal4 forward primer with extending HindIII and NotI sites ACGCACGCaagcttgcggccgcccaccATGAAGCTACTGTCTTCTATC and the Gal4 reverse primer with extending SalI site ATAGCTGCGTGCGTGCGTgtcgacttactctttttttgggtttgg. PCR products were digested with HindIII and SalI and ligated into pcDNA™3.1/Zeocin (Invitrogen, Carlsbad CA). pFR-eGFP, modified from pFR-Luc (Stratagene) was kindly provided by Rainer de Martin (University of Vienna).

For the construction of the inducible vectors (pH-TRE-Gal4, TRE-PR/Gal4 and pH-TRE-PRm/Gal4), a 7X Tet-Responsive Element (TRE) was amplified from the pTRE-tight (Clontech) with the forward primer with extending NruI site AGCTAGCTAGCTTCGCGAC ACGAGGCCCTTTCGTCTTCA and the reverse primer with extending BsrGI site CATTTTTTTCACTGCCTCGAGTGTACAAGCTAGCTAGCT. The PCR product was digested with NruI and BsrGI and cloned into the pH-CMV-eGFP vector (Gary Nolan, Stanford University) to replace the original CMV-eGFP cassette (creating pH-TRE). The forward Gal4 primer with extending BamHI site ATGCATGCggatccACCATGAAGCTACTGTCTTCTATC and the reverse primer with extending NheI site GCATGCATGCTAGCTTACTCT TTTTTTGGGTTTGG were then used to amplify the Gal4-based cassettes from pcDNA3.1-Gal4, pcDNA3.1-PRm/Gal4 and pcDNA3.1-PR/Gal4, which were then inserted into BamHI/NheI-digested pH-TRE. pBMN-i-mCherry was used for the creation of pBMN-i-rtTA. It was constructed by amplifying mCherry from pmCherry-C1 (Clontech) using the forward primer with extending NcoI site ATCGATGGATCCCCACCATGGTGAGCAAGGGCGAGGAG and reverse primer with extending XhoI site ATGGACGAGC TGTACAAGTAACTCGAGGATCGATC, and inserting it into partially digested pBMN-i-eGFP (Gary Nolan, Stanford University) with NcoI/SalI. pBMN-i-rtTA was then constructed by removing rtTA from the vector Tet-On® (Clontech) with EcoRI/BamHI and cloning it into pBluescript-SK (Invitrogen, Carlsbad CA), and subsequently removed with EcoRI/XhoI and ligated into pBMN-i-mCherry.pH-5xUAS-eGFP was constructed by digesting pFR-eGFP with MfeI/BsrGI and ligating the resulting 5xUAS-eGFP insert into pH-CMV-eGFP digested with MfeI/BsrGI to replace the CMV-eGFP cassette. ***Transfections***

For the transfection of HEK293T cells, 15µl of 2mg/mL Polyethylenimine linear 25kD (Polysciences, Inc.) and 3µg of each DNA was used. This mixture was added to cells grown in DMEM media (supplemented with 10% Fetal Calf Serum (FCS), Penicillin – Streptomycin (Pen-Strep), L-Glutamine) in a 10cm plate at ∼60–75% confluence. Cells were then analyzed by fluorescence microscopy and/or flow cytometry 24 hours post transfection.

### Production of Viral Particles for Retroviral Transduction

For the production of MLV based virus, the Phoenix GP cell-line (Nolan Lab, Stanford University, CA) at 50–60% confluence was transfected with 3µg of the packaging vector (pBMN-i-rtTA) and 3µg of a vector expressing the Envelope glycoprotein of the Vesicular Stomatitis Virus (pCI-VSVg). Media (DMEM with 10% FCS, Pen-Strep, L-Glutamine) was replaced 24 hours post-transfection and viral supernatant was collected 48 hours after transfection, filtered with 0.45 micron PTFE filters (Pall Corporation) and frozen at −80°C in 1mL aliquots. For the production of HIV based virus particles, HEK293T cells were transfected with 3µg packaging vector (pH vectors), 2µg pCI-VSVg, 1µg of Viral Protein R-expressing vector (pRSV-Vpr, shown to enhance viral titers) [35], and 3µg of pCMVΔ8.2, (kindly provided by Didier Trono, EPFL, Switzerland) [36]. Media (DMEM with 10% FCS, Pen-Strep, L-Glutamine) was replaced 24 hours post-transfection and viral supernatant was collected 48 hours post transfection.

### Transductions

SupT1 cells, grown in RPMI supplemented with 10% FCS, Pen-Srep, L-Glutamine, was treated with 5µg/mL Polybrene (Hexadimethrene Bromide, Sigma) and infected with viral stocks by centrifugation in a hanging bucket rotors centrifuge (Becton Dickinson) at 1500RPM, for 120 minutes at 32°C. Cells were then analyzed for expression at least 72 hours post-infection.

### Fluorescence Microscopy for Analysis of Expression

Cells were checked for fluorescence on a Zeiss Observer D1 microscope with a 50X lens connected to an AxioCam MRm camera, and analyzed on Axio-Vision software.

### Flow Cytometry and Sorting

Flow Cyometry was performed on a BD FACSAria with 405nm, 488nm and 633nm lasers. Data was collected on FACSDiva 6.1.1 software and then exported to FlowJo for analysis.
